# Ageing of skeletal muscle extracellular matrix and mitochondria: finding a potential link

**DOI:** 10.1080/07853890.2023.2240707

**Published:** 2023-08-29

**Authors:** Lubing Cai, Luze Shi, Zhen Peng, Yaying Sun, Jiwu Chen

**Affiliations:** a Department of Sports Medicine, Shanghai General Hospital, Shanghai Jiao Tong University School of Medicine, Shanghai, China; b Department of Sports Medicine, Huashan Hospital, Fudan University, Shanghai, China

**Keywords:** Ageing, sarcopenia, extracellular matrix, mitochondria, reactive oxygen species

## Abstract

**Aim:** To discuss the progress of extracellular matrix (ECM) characteristics, mitochondrial homeostasis, and their potential crosstalk in the pathogenesis of sarcopenia, a geriatric syndrome characterized by a generalized and progressive reduction in muscle mass, strength, and physical performance.

**Methods:** This review focuses on the anatomy and physiology of skeletal muscle, alterations of ECM and mitochondria during ageing, and the role of the interplay between ECM and mitochondria in the pathogenesis of sarcopenia.

**Results:** Emerging evidence points to a clear interplay between mitochondria and ECM in various tissues and organs. Under the ageing process, the ECM undergoes changes in composition and physical properties that may mediate mitochondrial changes via the systematic metabolism, ROS, SPARC pathway, and AMPK/PGC-1α signalling, which in turn exacerbate muscle degeneration. However, the precise effects of such crosstalk on the pathobiology of ageing, particularly in skeletal muscle, have not yet been fully understood.

**Conclusion:** The changes in skeletal muscle ECM and mitochondria are partially responsible for the worsened muscle function during the ageing process. A deeper understanding of their alterations and interactions in sarcopenic patients can help prevent sarcopenia and improve its prognoses.

## KEY MESSAGES

Sarcopenia is a senile syndrome featured by a progressive and generalized decline of muscle mass, strength, and physical performance. Given the complexity and importance of the extracellular matrix (ECM) and mitochondria of skeletal muscle, we, in this review, summarized current progress in the alterations of ECM properties and mitochondrial homeostasis in aged skeletal muscle, and have found several potential links between them. And we believe that this work could provide new insight into the prevention and treatment of age-related sarcopenia.

## 1. Introduction

Over the past century, we have witnessed the remarkable advances achieved in medicine, with the estimated life expectancy nowadays increasing rapidly. Nonetheless, the quality of life of some longevous in their later years often cannot meet satisfactory, one apparent reason is the deterioration in skeletal muscle structure and function during ageing.

Skeletal muscle is the largest proportion of tissues in the human body with complete regeneration ability. Muscle fibres form bundles, which form muscle tissue, and each layer is sequentially wrapped by the extracellular matrix [1]. Type-I and type-II fibres, also referred to as slow and fast fibres, respectively, are the two main categories of muscle fibres. Type-I fibres are known as slow oxidative fibres, while type II are fast oxidative/glycolytic fibres [2]. Muscle fibres, together with various types of mononuclear cells, are located in the skeletal muscle microenvironment [3]. Under the ageing process, the fast-twitch oxidized myofibers decreased by 40% [2]. In addition, while the histochemical staining of young muscle exhibits a ‘mosaic’ appearance that different fibres are interspersed, the older muscle presents a ‘patchy’ appearance due to the cluster of individual myofiber types [4].

The term sarcopenia referred to a generalized skeletal muscle disorder characterized by a gradual decline in lean body mass and function. It occurs commonly during the age-related process and increases the risks of falls, physical disability, hospitalization, and mortality [5–7]. Besides age-associated primary sarcopenia, chronic diseases such as malignant cancer, COPD, heart failure, renal failure, and others can also lead to secondary sarcopenia [8]. According to the clinical definition of the European Working Group on Sarcopenia in Older People (EWGSOP) and the Asian Working Group for Sarcopenia (AWGS), its prevalence is estimated to be 10–30% across the globe [9], making it a public health issue with serious social and economic consequences.

Mitochondria, as fundamental organelles for cell survival, play a central role in respiratory ATP production, apoptosis, calcium buffering, and the generation of reactive oxygen species (ROS) [10]. Mitochondria are critical for preserving muscle mass volume and function throughout the lifespan [11], and mitochondrial dysfunction is one of the hallmarks of ageing [12]. The ageing-associated alterations in skeletal muscle mitochondria include morphology, components, dynamics, biogenesis, and mitophagy [13]. For instance, the ability of mitochondrial ATP production declined in elder adults [14], and the accumulation of impaired mitochondria may play a part in muscle fibre death [15]. Further, mitochondria are one of the major sources of ROS. As age advances, the levels of ROS increase, enhancing DNA damage, protein oxidation, and mitochondrial dysfunction [16], subsequently further increasing ROS production. A favoured hypothesis of sarcopenia pathobiology regards mitochondrial dysfunction as a central onset factor [17].

As the main part of the supporting structure of this microenvironment, extracellular matrix (ECM) represents 10% of skeletal muscle weight [18]. ECM mainly consists of protein, polysaccharides, and RNA [19]. Proteins can mainly be further divided into collagens, non-collagenous glycoproteins, and proteoglycan (PG) [20]. These contents participate in countless biological processes, such as the myogenic process of skeletal muscle progenitors, quiescence maintenance of satellite cells (SCs) [21], and the process resulting in myoblast-to myotube-fusion [22]. ECM helps maintain the shape and biomechanics of skeletal muscle. In adult skeletal muscle, the majority of the collagen fibres are aligned perpendicular to the direction of the myofibres no matter in young or old bodies. In contrast, decreased tortuosity can be observed in old ECM, as compared to that observed in young muscle. Moreover, ECM can act as a mediator to transmit mechanical loading [23]. Thus, all these properties can influence the homoeostasis of skeletal muscle.

**Figure 1.**
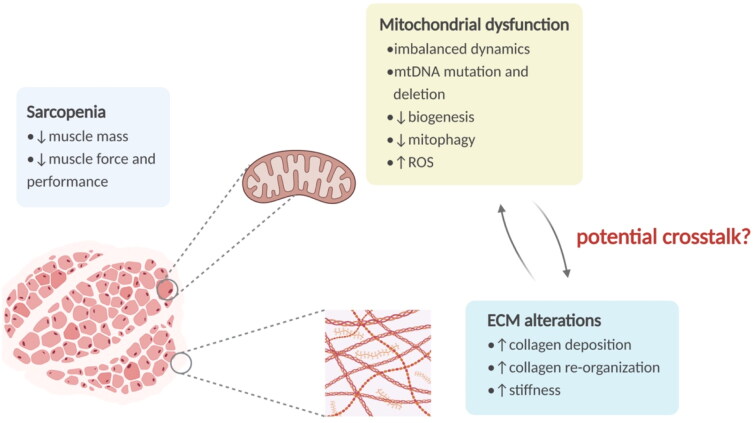
The overview of sarcopenia and relevant alterations in mitochondria and ECM. Sarcopenia is a senile syndrome featured by a generalized decline of muscle mass, strength, and physical performance. Mitochondria in skeletal muscle show various age-associated alterations, including mtDNA damage, imbalance of dynamics, biogenesis decline, mitophagy impairment, and ROS overproduction. And the aged ECM exhibits increased collagen deposition and re-organization, leading to a stiffer matrix. ECM: extracellular matrix; mtDNA: mitochondrial DNA; ROS: reactive oxygen species.

Given the complexity of ECM of skeletal muscle, we, in this review, summarized current progress in ECM properties and the relationship between these properties and mitochondrial homeostasis in skeletal muscle, with a specific focus on ageing (Figure 1).

## 2. ECM with ageing skeletal muscle

### 2.1. ECM structure and composition

Typically, the ECM of skeletal muscle tissue can be divided into three subdivisions: the epimysium is the dense connective tissue that envelops the entire muscle; the perimysium derives from the surface layer and surrounds the muscle bundle; the innermost layer enclosing each muscle fibre is the endomysium, also known as the basement membrane [24]. The epimysium mainly contains type I, III collagen, and fibronectin [25]; while the perimysium contains collagen (type I, III, V, and VI), proteoglycan, fibronectin, etc. [26]; the endomysium contains primarily type I, III, and V collagen, laminin, fibronectin, and others [27]. The ECM consists of about 300 proteins, called the core matrisome, including proteins such as collagen, PGs, and glycoproteins [28]. There are two main types of ECM with different locations and compositions: the interstitial connective tissue matrix, which surrounds the cells and provides a structural scaffold for the tissue; and the basement membrane which separates the epithelium from the surrounding matrix.

The thickest and strongest of the structure of ECM, so-called the epimysium, covers the whole muscle [29]. The tendon that joins the muscles to the bones continues as the epimysium [30]. And the epimysium contains the main blood vessels and nerves that nourish the muscles [31]. There are many myofibres that surround the perimysium to form fascicles, which are connected to the epimysium [32]. The perimysium contains larger blood vessels and nerves and transmits the force to tendons [33]. Each muscle fibre is encircled by a thin and delicate component of the ECM mysium, called endomysium. Small arteries and neurons are found in the endomysium, which also plays a significant role in myogenesis and muscle regeneration and preserves the integrity of the muscle [34].

### 2.2. ECM function

ECM serves as the scaffold for cell–matrix interactions, which is essential for many physiological activities within skeletal muscle tissue, where ECM provides the microenvironment that promotes cell adhesion, migration, proliferation, and differentiation. Moreover, the ECM characteristics are also impacted by the physiological functions of skeletal muscle [35]. Skeletal muscle development is aided by the way ECM and skeletal muscle cells interact to adapt muscle cells to their microenvironment. Muscle aging is significantly influenced by the ECM [19]. In old bodies, skeletal muscles typically represent fibrotic morphology [36]. The orientation of healthy collagen fibres declines with age and is replaced by an irregular fibre network with lessened crimp production, which is a hallmark of age-associated muscle fibrosis [37]. And there is an increase in collagen content and cross-linking of collagen fibres [38]. Therefore, the elastic modulus of the ECM can be increased about 35-time in old muscles [39]. The deposition of basal lamina proteins can drive SCs out of their niches, affecting the regulation of their divisions [36]. Due to this, the number of SCs in old muscles typically decreases when compared to young muscles [40], which is a main alteration in skeletal muscle ageing [12,41]. In addition to its effects on SCs, a dysregulated basal lamina is anticipated to hinder muscle regeneration [42].

Collagen is the most abundant component of the ECM in the skeletal muscle. In the skeletal muscle, there are three distinct types of cells that can secrete collagen, exactly fibroblasts, fibroblast/adipose progenitors, and SCs [43]. Collagen presents numerous fibre bundles in the ECM and is classified into several subtypes. Type I, III, V, and XI are the main types of collagen fibers in skeletal muscle [44]. Type VI is the microfibrins that form a filamentous grid. Type IV and type VIII are important components of the basal layer. Type XXII is located at the tissue junction and helps to stabilize the junctions and skeletal muscle tendon adhesion [45]. Type-VI collagen is a major component of the ECM that regulates the function of SCs and maintains them during muscle regeneration. Besides, there are positive interactions between SCs and fibroblasts during muscle regeneration after injury [46]. It has been shown that increased collagen concentration leads to increased skeletal muscle stiffness and decreased mechanical properties but protects smaller muscle fibres from damage [47].

Type-I collagen confers tensile strength and rigidity to the muscle significantly, while in the process of skeletal muscle growth, it inhibits myogenic differentiation. The expression level of type-I collagen is downregulated during myoblast differentiation, while an increase in extracellular space has been observed with age [47]. Other studies have shown that type-I collagen may contribute to the proliferation and migration of myoblasts [48,49]. Furthermore, Leduc-Gaudet et al. demonstrated that aged skeletal muscle can be revitalized by attenuating the collagen I deposition [50].

Type-IV collagen, a major component of the basal layer, can promote IGF1-mediated myoblast migration, differentiation, and fusion, thereby promoting skeletal muscle regeneration [51]. Mutations in the Col4α1 gene can cause skeletal muscle atrophy. Type VI is a key component of the satellite cell niche [52], regulating SC function and maintaining the SC pool [36]. The deficiency of type-VI collagen has been shown to influence the processes of differentiation and regeneration, causing disturbed muscle function, protein dysfunction, mitochondrial dysfunction, autophagy dysfunction, and esterification of microtubule-associated proteins, leading to premature aging of skeletal muscle and severe myopathic [3,36,52].

### 2.3. ECM-related pathways

In skeletal muscle, the ECM plays a major role in determining the stiffness of the entire microenvironment. Excessive accumulation of ECM in the aging skeletal muscle microenvironment leads to increased stiffness, which inhibits the myogenic differentiation capacity of SCs [53]. For example, in the ECM, the addition of fibronectin can induce the formation of ordered myotubes [54], while that of collagen may cause disturbances in the myotube sequence. The ECM components required for maintaining the satellite cell niche are upregulated in the skeletal muscle of young mice compared to aged mice [55]. The synergistic effect of ECM stiffness and WNT 7a can regulate the symmetric division of the SCs [56], thereby affecting the fate of the satellite cells.

Mechanical stimulation regulates the availability of secreted signalling factors, which is followed by mechanoregulation processes such as ECM deposition, rearrangement, and removal to maintain overall form and function [57]. The mechanical homoeostasis which involves ECM and its receptors, such as collagen and elastin, transmits mechanical loads; the transmembrane receptors, mainly integrins [58], actin filaments, non-muscle myosins, and related proteins, constitute the cytoskeleton and transmit mechanical loads and signals within the cell [3].

The key cellular structures involved in mechanical loads are cell surface receptors that enable cells to attach to the ECM or other cells [59]. In this case, the key mechanosensors are integrins in focal adhesions and cadherin in adhesion junctions [60,61], interacting with intracellular signalling molecules and physically linked to cytoskeletal actin through a series of junction proteins [62,63]. The delivery of mechanical cues within the cell enables the conversion of mechanical forces into biochemical signalling. These mechanisms are essential for cells to probe and measure the mechanical properties of their microenvironment, including ECM stiffness, attachment to other cells, the stretching of the tissue or the shear stress exerted by the flowing fluid, and for enhancing the appropriate response to these cues. Key signalling pathways associated with integrin activation include the RHO-related protein kinase (ROCK) and the mitogen-activated protein kinase (MAPK) pathway [64].

### 2.4. Collagen deposition and re-organization in ageing skeletal muscle

Old skeletal muscles are characterized by reduced muscle mass [65]. The half-life of skeletal muscle collagen increases with age [66]. Specifically, total collagen and relative collagen content increases by 35–40% of the extracellular space and is associated with a higher proportion of type-I collagen to type-III collagen [67]. In the elderly, type-IV collagen accumulates in the basal layer of the slow-twitching muscle, while the laminin concentration appears to decline [55]. Increased basal protein deposition also shows the shedding of SCs from their niche, which negatively affects the regulation of satellite cell division [40]. In old age, fibronectin is depleted from the basal layer, reducing the integrin attachment points in SCs [68]. Laminin in the fast-twitch muscle increases with age, while those in the slow-twitching muscle depletes. As another component of the basal layer, type-IV collagen forms a network and serves as a scaffold protein for laminin and other cell-anchored proteins [69]. Ageing increases type-IV collagen levels while reducing the ability of muscles to regulate type-IV collagen, thereby hindering the assembly of fibronectin with collagen type IV [70]. Type-VI collagen, a bridge between basal and reticular layers, is abnormally increased in old age, disrupting the regular structure of the layers [71]. Finally, the increase of aberrant glycosylation cross-links hardens ECM, dysregulating quiescent SCs and muscle force delivery [38].

The myogenic capacity of SCs In ageing muscle decreases, while fibrogenic ability is enhanced [72]. Age-related alterations of the composition in the ECM and the Wnt-signalling pathway regulate this myogenic fibrotic transition. Wnt/catenin expression is upregulated by transforming growth factor-β (TGF-β) signalling and vice versa [73]. It has already been shown that TGF-β is upregulated in aged myocytes and fibroblasts, with increased levels of phosphorylated Smad 2/3, β-catenin, and collagen deposition [74]. Thus, collagen deposition in intact muscles increases with age. In old bodies, skeletal muscle usually exhibits a fibrotic pattern [75], as characterized by a loss of a clear bidirectional lattice orientation of healthy collagen fibres and abundant in unstable fibre webs with reduced coil formation [76]. In addition, the absolute collagen content and non-enzymatic crosslinking of collagen fibres may increase. Thus, the elastic modulus of the ECM can increase approximately from 12 ∼ 418 kPa for young to old muscles, an effect that is driven by the accumulation of densely packed and widely cross-linked collagen [39].

### 2.5. The alteration in the progression of sarcopenic obesity

Sarcopenic obesity (SO), defined as the coexistence of sarcopenia and obesity, is a systemic aggregation of metabolic disorders caused by multiple causes and also closely associated with ageing [77]. Sarcopenia, it is a systemic aggregation metabolic disorders from multiple causes, a vicious cycle formed between reduced physical activity/sarcopenia – insulin resistance – inflammation/oxidative stress – lipotoxicity [78–80], a process of the metabaging cycle [81]. Each factor has an independent effect on the mass and quantity of muscle and fat, while their crosstalk has a greater impact on body composition change: decreasing muscle mass and strength as well as increasing fat mass [82].

Both ageing and physical inactivity contribute to weight gain, along with an increase in adipocyte size. Obesity can activate macrophages, mast cells, and T lymphocytes [83]. These immune cells, together with adipocytes, secrete extreme adipokines, such as leptin, and cytokines, such as tumour necrosis factor-α (TNF–-α), interleukins (iLs), interferon–-γ (INF–-γ), causing low-grade inflammation [83–85], leading to imbalanced myokine production and mitochondrial dysfunction. Mitochondrial dysfunction leads to more lipid peroxidation, thereby strengthening the collection of lipid intermediates and reactive oxygen metabolites, accelerating the vicious cycle [86]. Both accumulation of damaged mitochondria and abnormal metabolites result in the apoptosis of motor neurons and muscle fibres [87,88].

## 3. Mitochondria with ageing skeletal muscle

One major feature of skeletal muscle is that it can respond to endogenous and exogenous physiological stimuli by changing its contractile machinery and energy metabolic capacity [89]. Hence, it contains a large number of mitochondria, and a specialized skeletal muscle mitochondrial system has been evolved to match its high energy consumption.

### 3.1. The classification of mitochondria in skeletal muscle

Generally, according to their subcellular location, skeletal muscle mitochondria can be classified into subsarcolemmal (SS) mitochondria and intermyofibrillar (IMF) mitochondria. The SS group is normally located underneath the sarcolemmal membrane. They tend to be globular, with few branches [90], and account for about 20% of the total mitochondria of skeletal muscle [91]. On the other hand, the IMF group is characterized by residing within the intermyofibrillar spaces, mostly situated at the I-band of the sarcomere [92], with generally elongated tubular shapes [90], representing the remaining 80% of mitochondria within skeletal muscle [91]. Besides localization and morphology, the two subpopulations also differ in composition, energy metabolism, and functions [93,94].

In recent years, a new classification of mitochondria is proposed based on three-dimensional high-resolution electron microscopy. Researchers classified the skeletal muscle mitochondria into four populations: paravascular mitochondria, I-band mitochondria, fibre parallel mitochondria, and cross-fibre connection mitochondria. Though using a novel classification, the study also supports the previous findings that the membrane potential alternation propagating via the united mitochondrial reticulum provides a critical pathway for skeletal muscle energy distribution [95].

### 3.2. Mitochondrial morphology in ageing skeletal muscle

In skeletal muscle, an increased abnormal rate can be found in aged mitochondria, which can be characterized by a swollen, enlarged [96–98], and more branched [98] morphology and/or structurally impaired, for example, disrupted membrane, loss of cristae, matrix dissolution, and vacuolization [99,100]. Some observed that mitochondria from aged mice often extend longitudinally between myofibrils [96], while others noticed a greater mitochondrial fragmentation, especially the decrease of SS mitochondria [101]. The maximal depth of SS mitochondria and the average area of individual IMF mitochondria also decline with age [102].

### 3.3. Mitochondrial dynamics in ageing skeletal muscle

Mitochondria networks are highly dynamic that undergo continuous fission and fusion events, the balance of which is fundamental in maintaining a normal mitochondrial morphology and adapting to the energy requirements [11]. Mitochondrial fusion refers to the process that mitochondria coordinately integrate their outer and inner membranes, leading to an elongated single one [11,89]. By allowing material supplementation between individual mitochondria, this process helps maintain the self-homeostasis of this organelle [103]. Conversely, fission describes the scission processes of both outer and inner membranes of mitochondria [104] so that the tubular networks get fragmented into smaller organelles [11,89]. Fission is required to efficiently remove the dysfunctional mitochondria from the network [11], enabling mitophagy to eliminate damaged components [92,105], and inducing cell death [92].

Morphological alterations in aged muscles from mice, rodents, and humans have been demonstrated to be dependent on impaired machinery coordinating mitochondrial dynamics. An age-dependent loss of the expressions of fission and fusion genes can be observed in both mice [100,106] and humans [106,107]. Fusion-defective mice displayed an age-associated extensive ROS production, reduced skeletal muscle mitochondrial respiration, and a more significant reduction in muscle cross-sectional area [100]. Consistently, overexpression of fusion genes can reversely protect mice skeletal muscle from atrophy [11]. Moreover, the deficiency in mitochondrial dynamics contributes to greater production of ROS, and oxidative stress could in turn disturb mitochondrial dynamics [108]. These researches highlight the importance to establish balanced mitochondrial dynamics for maintaining muscle homoeostasis.

### 3.4. mtDNA mutations and deletion in aging skeletal muscle

mtDNA, the own genetic materials in mitochondria, are a bunch of short [109] and closed circle double-stranded DNA [110], uniparentally inherited from the mother [109]. mtDNA encodes the mitochondrial respiratory complexes localized on the inner mitochondrial membrane, all of which are critical polypeptides for the generation of ATP [111].

The mtDNA copies in human skeletal muscle samples gradually decline with age [112], while mtDNA mutations accumulate progressively [87,113,114] and are associated with sarcopenia [115,116]. The accumulation of mtDNA mutations in skeletal myocytes can result in fibre atrophy, breakage, and loss [117] and can negatively affect the assembly of mitochondrial electron transport chain complexes, which later contributes to a decrease in oxidative phosphorylation [118], ultimately enhancing muscle apoptosis [87]. It should be noted that mitochondrial dynamics are important for mtDNA stability and fidelity [119].

### 3.5. Mitochondrial biogenesis in ageing skeletal muscle

Healthy skeletal muscle has the ability to generate new organelles via mitochondrial biogenesis, and this ability declines with age [120]. The biogenesis induction is correlated with a master coactivator, peroxisome proliferator-activated receptor-γ coactivator-1α (PGC-1α). It plays a crucial role in mtDNA homoeostasis [11,121,122], ROS scavenging, and fibre-type balance [123].

The expression of PGC-1α is enriched in skeletal muscle [121]. Its reduction in the aging process [124–127] is associated with a decrease in mitochondrial function [124]. Loss of PGC-1α expression contributes to muscle atrophy [128] and chronic systemic inflammation [129] associated with ageing. PGC-1α overexpression in mice skeletal muscle showed decreased muscle fatigability and promoted muscle oxidative capacity [123,130]. In response to exercise, PGC-1α transcription and activity are up-regulated in skeletal muscle [131], resulting in increased mitochondrial biogenesis and promoted mitochondrial enzyme activities to counteract sarcopenia [132].

### 3.6. Mitophagy in ageing skeletal muscle

In addition to mitochondrial biogenesis, the mitochondrial quality control system cannot function without mitophagy, an opposing process to remove dysfunctional organelles as a specific form of autophagy. Once the mitochondria become depolarized under stressed or impaired conditions, autophagosome is formed and fuses with lysosome [109], thereby degrading the mitochondrial fragments.

Mitophagy is required to maintain muscle mass and myofiber integrity. Specifically knocking out the autophagy gene results in muscle loss, weakness, and accumulation of atypical swollen mitochondria [133]. Although mitophagy mediators of aged skeletal muscle may be up-regulated [134,135] or down-regulated [50,89,136], there is a consensus that, with ageing, mitophagy is attenuated [134,137] and is poorly responsive to exercise [134]. In the light of this belief, mitophagy might be an attractive therapeutic target for improving skeletal muscle health and performance.

### 3.7. Influences of endocrine stimuli to mitochondria and sarcopenia

Insulin is fundamental for achieving a positive protein balance in skeletal muscle [138]. Insulin resistance not only drives sarcopenia via redistributing the ECM as we discussed above, but also through impacting muscle mitochondrial protein synthesis [139,140]. Studies have suggested that insulin could promote the activities of mitochondrial enzymes, ATP production, and mRNA concentrations of genes encoding mitochondrial proteins, facilitating translation, partially *via* PI3K and mTOR signalling pathways [140].

As an endocrine tissue, skeletal muscle has the ability to generate a number of signalling molecules known as myokines [141], which can affect other tissues and organs through autocrine, paracrine, and endocrine pathways [138]. Myokines trigger effective stimuli for mitochondrial biogenesis in skeletal muscle. Specifically speaking, in sarcopenic people, decreases in interleukin-15 (IL-15), insulin-like growth factor hormone (IGF-1), and irisin can be observed, while the level of myostatin, interleukin-6 (IL-6), and tumour necrosis factor-α (TNF-α) increase [142].

IL-15 is a well-known cytokine that works through the JAK/STAT cascade, PI3K/Akt and AMPK-signalling pathway. It is highly expressed in skeletal muscle and plays a role in enhancing muscle hypertrophy in response to physical exercise [143]. In muscle and serum samples from elderly mice, IL-15 levels decline with ageing [144]. Chronic IL-15 treatment results in a greater mtDNA copy number [145]. Furthermore, increased levels of circulating IL-15 are linked to modulating mitochondrial activity, by promoting the expression of several downstream proteins such as PGC1α, PPARs, and SIRT1 [146].

Irisin is a cleaved product of fibronectin type-III domain-containing protein 5 (FNDC5) and the muscles have been regarded as one of its major sources [147]. It promotes mitochondrial biogenesis via activating the AMPK-PGC-1α signalling, thus increasing cellular oxygen consumption, metabolic rate, and mitochondrial content in muscles [148]. In comparison to the normal group, the sarcopenic group has reduced circulating irisin levels [149]. Irisin treatment in mice helps against muscle atrophy by upregulating the insulin-like growth factor-1 (IGF-1)/Akt/mTOR pathway, as well as decreasing myostatin gene expression [142], thus improving muscle regeneration and inducing hypertrophy [150]. Additionally, it is hypothesized that myostatin expression in skeletal muscle may be decreased by irisin-induced ERK activation and a concurrent rise in IGF-1 expression [151].

Sirtuins are NAD+-dependent deacetylases that serve a crucial regulatory role in cellular processes such as DNA repair and metabolism [152]. As a member of the sirtuin family, SIRT1 activates the downstream PGC-1α by deacetylation, increasing the production of mitochondrial proteins and genes involved in β-oxidation [153]. Oyster hydrolysates administration could significantly stimulate the SIRT1/PGC-1α signalling, regulating protein turnover and mitochondrial biogenesis, and therefore attenuate muscle atrophy, increasing both grip strength and exercise endurance [154].

Myostatin, a member of TGF-β superfamily, negatively regulates skeletal muscle growth [142]. Studies have regarded myostatin as one of the potential biomarkers of muscle wasting [155,156], with sarcopenic patients exhibiting higher myostatin gene expression, as well as circulating myostatin concentration rises with age [155]. Myostatin impacts both mitochondrial biogenesis and mitochondrial dynamics via modulating Smad pathways and the expression of Drp1 and Fis1 [153,157]. In human skeletal muscles, an inverse correlation could be observed between myostatin expression and insulin sensitivity [158], by which the higher expression of myostatin in the elderly may also impair mitochondrial biogenesis.

### 3.8. ROS with ageing skeletal muscle

Reactive oxygen species (ROS) consist of radical superoxide (O2−) and hydroxyl radicals (HO •), as well as nonradical hydrogen peroxide (H2O2) [159]. The sources of ROS production in skeletal muscle include mitochondria, nicotinamide adenine dinucleotide phosphate (NADPH) oxidases, and Phospholipase A2 (PLA2) dependent processes [160].

ROS is a double-edged sword. Physiological levels of ROS are involved in cell signal transduction pathways, such as promoting cell proliferation and differentiation. On the other hand, overproduced ROS can cause the oxidation of DNA, proteins, and lipids, stimulating oxidative stress and further lethal cascades [16,161,162]. Mitochondrial ROS generation is increased during ageing in skeletal muscle [163,164]. The increase in ROS production and correlated redox signalling have been proven to play a key role among the pathophysiological mechanisms leading to sarcopenia [165]. The excessive ROS in ageing muscles may downregulate the PI3K-Akt axis to induce muscle atrophy [166]. High levels of ROS suppress the myofibrillar Ca^2+^ sensitivity, accelerate muscle fatigue [167], and leading to the senescence of SCs [168]. ROS-induced oxidative stress, together with chronic inflammation, can also restrain the SCs from being activated, undermining muscle regeneration [16]. Besides, ROS over-production at neuromuscular junctions (NMJs) decreases neurotransmitter release, which may impair the generation of the action potential, contributing to sarcopenia [169,170].

In aged muscles, the level of the components of antioxidant defense systems, including copper‑zinc-superoxide dismutase (CuZnSOD), manganese superoxide dismutase (MnSOD), and glutathione peroxidase (GPX), are decreased compared to the young [171]. Mice lacking cytoplasmic CuZnSOD, the major antioxidant enzyme, exhibit a dramatic exacerbation of age-dependent skeletal muscle atrophy, increased production of pro-inflammatory cytokines [172], partial denervation, degeneration of NMJs, and mitochondrial dysfunction [160]. Meanwhile, the CuZnSOD-deficient mice show a greater loss in muscles containing predominantly type-II fibres, a well-recognized histological characteristic of sarcopenia [173].

Collectively, these data illustrate a clear link between increased ROS production and age-associated sarcopenia. The overproduction of ROS contributes to oxidative stress, exacerbating mitochondrial dysfunction and accumulation of inflammatory cytokines, leading to sarcopenia during aging, which further initiates oxidative stress, forming a vicious cycle [16].

## 4. The interplay between mitochondria, ROS, and ECM

**Figure 2.**
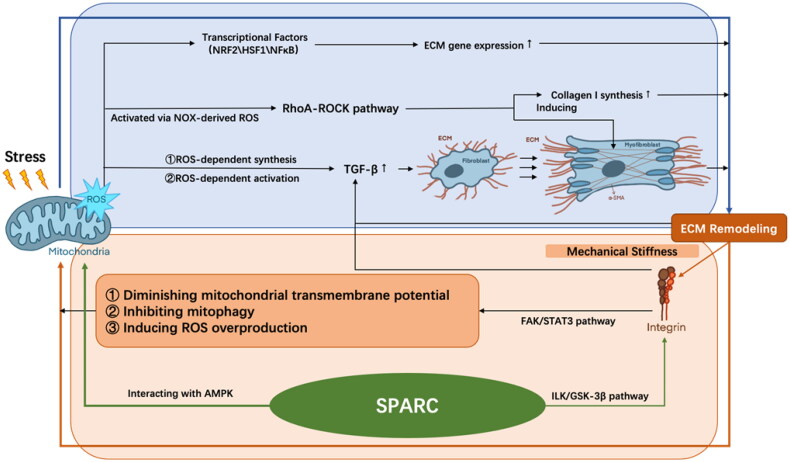
The interplay between mitochondria and ECM during aging. The excessive ROS may contribute to the mechanical tension in stiffer ECM scaffolds through modulating transcription factors, activating RhoA-ROCK pathway, and releasing TGF-β. SPARC can induce ROS generation and activate integrin-linked kinase, contributing to muscle ECM remodelling, altogether differentiating fibroblasts into myofibroblasts, and is further responsible for ECM accumulation in a vicious cycle. ROS: reactive oxygen species; NRF2: nuclear respiratory factor 2; HSF1: heat shock factor 1; ECM: extracellular matrix; α-SMA: α-smooth actin; FAK: focal adhesion kinase; STAT3: FAK-signal transducer and activator of transcription 3; SPARC: secreted protein acidic and rich in cysteine; AMPK: adenosine monophosphate-activated protein kinase; ILK: integrin-linked kinase; TGF-β: transforming growth factor-β.

As discussed above, ECM and mitochondria are both known to play a critical role in the development of sarcopenia. So far, emerging evidence, either direct or indirect, has revealed that alterations in mitochondria, especially ROS, can affect ECM and vice versa (Figure 2).

Fibrosis in the aged skeletal muscle is characterized by excessive ECM protein production and deposition, which leads to alterations in mechanical tensions. Since mitochondria are embedded into the cytoskeletal framework and interact with actin filaments, abnormal mechanical stress absorbed by the cell cortex that consists of actin and actomyosin fibres will then result in oxidative stress, ROS formation, and decreased ATP production [174]. Increased ROS production and antioxidant reduction can lead to disorganization of ECM and extra-intracellular tension formation [174]. Firstly, ROS modulates the activity of transcription factors involved in the gene expression of ECM, such as nuclear respiratory factor 2 (NRF2), HSF1, and NFκB [175]. Secondly, ROS controls the release of growth factors. TGF-β1 is one of the most well-recognized profibrogenic mediators [176], which is synthesized with a ROS-dependent mechanism and can be activated by releasing from its inactive complex through the mediation of ROS [177]. It later causes fibroblast differentiation into myofibroblasts, which enhances matrix tension. TGF-β1 can also bind to thrombospondin-1 (TSP1), thereby stimulating fibroblasts and further deteriorating ECM fibrotic. In addition, NADPH oxidase (NOX)-derived ROS directly activate the Ras homolog gene family, member A (RhoA)-ROCK pathway, which regulates ECM accumulation via inducing α-smooth actin (α-SMA), a myofibroblast differentiation marker, and collagen-I synthesis [178]. Furthermore, a distorted cytoskeleton directly affects mitochondrial respiration in fibroblasts and sensitizes the organelle to further energetic insult [179]. In cardiac myocytes, baseline metabolism is influenced by ECM stiffness, with basal respiration and ATP production increasing with greater elastic modulus [180]. And their mitochondrial network architecture is associated with the alterations in ECM rigidity. For cells with low- and high-aspect ratio, the mitochondrial network became smaller and more fragmented as ECM rigidity increased [181], which produces an excessive amount of ROS [182]. These influences of the mechanical tension in stiffer ECM scaffold, together with cytokines such as TGF-β, could differentiate fibroblasts into myofibroblasts [183] and is further responsible for ECM accumulation in a vicious cycle.

Moreover, ECM stiffness affects the mitochondrial cycle via the integrin’s cytoskeleton complex. Integrins are heterodimers of non-covalently associated α and β subunits, with α subunits regulating the recognition of the cation-dependent ligands, and β subunits regulating intracellular skeletal interactions and signalling [184]. The integrins bind ECM proteins to the actin cytoskeleton, acting as a matrix receptor that allows direct transfer of the mechanical stiffness [185]. Focal adhesion kinase (FAK) is one of the major integrin signalling mediators. αVβ6 integrin has been implicated to be able to activate the FAK-signal transducer and activator of transcription 3 (STAT3) pathway, which induces the mitochondrial equilibrium impairment and ECM gene expression responsible for fibrosis [174]. Additionally, the upregulated αVβ6 integrin is responsible for the activation of TGF-β, inducing ECM [186] and ROS overproduction, diminishing mitochondrial transmembrane potential [187], and inhibiting mitophagy through decreasing mitophagy regulators such as PTEN-induced putative kinase 1 (PINK1) and Parkin [174].

Similar to the progressive rigidity of aged muscles, the tumour stroma is also known to be characterized with progressive ECM deposition and stiffness during tumour promotion [188]. In a mouse model of metastatic breast cancer cells, Romani et al. [189] elucidated that a soft ECM promotes Drp1-mediated mitochondrial fission and an NRF2-dependent antioxidant response. Tharp et al. [190] demonstrated that the physical properties of the extracellular microenvironment could alter mitochondrial morphology in human mammary epithelial cell cultures. Solute carrier family 9 member A1 (SLC9A1) serves a transduction role of the matrix stiffness signal at the adhesion sites, increasing mitochondrial calcium concentration and ROS production, and ultimately, this overproduction of ROS further leads to morphological and metabolic alterations in mitochondria in turn.

For muscle cells, the secreted protein acidic and rich in cysteine (SPARC), a matricellular protein implicated in fibrillar collagen assembly in the ECM [191], has been reported to be associated with some chronic pathologies caused by ageing [192]. SPARC was suppressed in the gastrocnemius muscle of old mice [193], which was indicated to cause myofiber atrophy [194]. Melouane et al. demonstrated that the addition or inhibition of SPARC in C2C12 myoblasts leads to increased or decreased expression of mitochondrial proteins, respectively [195]. Mechanistically, SPARC regulates ECM and mitochondria through two different pathways. On the one hand, SPARC activates integrin-linked kinase (ILK), thereby phosphorylating and inactivating glycogen synthase kinase-3β (GSK-3β), contributing to muscle ECM remodelling. On the other hand, it modulates mitochondrial protein expression through direct interaction with adenosine monophosphate-activated protein kinase (AMPK), which then modulates the production of ROS [196]. Meanwhile, inhibited SPARC could induce or suppress ROS production during various tumorigenesis [197–199], and the expression of SPARC is also positively correlated with greater oxidative stress [200]. These studies have suggested that SPARC is an important bridge between ECM and ROS.

Chronic fatigue syndrome (CFS), also known as myalgic encephalopathy (ME), is a debilitating medical condition whose mechanisms and diagnosis are still controversial and with great heterogeneity [201]. It is characterized by chronic unexplained fatigue, post-exercise malaise, and a variety of multi-system symptomatology, all of which are partially linked to the alterations in mitochondrial function, influencing crucial proteins like the pyruvate dehydrogenase complex, AMPK, and mTOR proteins [202]. When compared with healthy control subjects, CFS patients also have reduced levels of antioxidants and ATP production, and increased levels of peroxides and superoxide [203]. Meanwhile, insulin resistance may also occur in CFS patients. Given that the high level of insulin could favour fat deposition [203] and its interaction with mitochondria as we have mentioned before, it is probable that the CFS shares some similarity with sarcopenia. However, CFS also implicates other tissues and organs, such as the central nervous system and the hypothalamus–pituitary–adrenal axis [201]. More in-depth studies are still needed.

Combing resistance and endurance training has been demonstrated to reduce myostatin in sarcopenic elderly [204]. The SIRT1-AMPK-PGC1α pathway and genes involved in mitochondrial biogenesis, which decreases during aging, are attenuated during exercise [142,205]. The increased level of AMPK after exercise also leads to promoted mitophagy [206]. A single bout of exercise could induce SPARC secretion from myocytes [207]. It has been demonstrated that resistance exercise significantly increases IGF-1, which has a strong correlation with the generation and strength of muscles and even impacts insulin sensitivity in the elderly with sarcopenic obesity [208]. Additionally, endurance exercise and resistance exercise enhance Mfn2 and Drp1 expression, modulating the balance of mitochondrial dynamics in aged rats [206,209]. However, resistance training should be done with caution, as it may increase the risk of injury, especially for the elderly. The effects of physical exercise also differ depending on the type of activity.

Calorie restriction, as a common diet strategy, has been reported to initiate mitophagy in various tissues, including skeletal muscles. It can also suppress pathways associated with inflammation and ROS while potentiating AMPK-SIRT1 signalling [210]. Nevertheless, calorie restriction seems to have no effect on skeletal muscle mass [211]. Another popular dietary plan is intermittent fasting, which has been acknowledged to boost skeletal muscle mass as well as lessen body fat, via modulating gut microbiota, and combating the oxidative and inflammation conditions [212,213]. What is more, a diet with high-protein intake and essential amino acid supplementation is able to lower fasting glucose, improve insulin responses, and is crucial for the synthesis of muscle protein [214]. In aged mice, improving muscle strength and physical performance is achieved by lowering serum phosphate levels through dietary phosphate restriction or phosphate binders administration. The expression of atrogin‐1 in old animals fed with the low‐phosphate diet is lower. In addition, skeletal muscle fibrotic regions in aged mice fed with a low-phosphate diet showed a considerable reduction, suggesting that age-related fibrosis could be delayed by a decreased serum phosphate concentration [215].

The above evidence showed that physical exercise and diet administration independently helps ameliorate sarcopenia through modulating both mitochondria and ECM. Moreover, the whole is greater than the sum of the parts. Net muscle protein balance remains negative when resistance exercise is performed in the fasted state, and the muscle protein synthesis rates after combining exercise and amino acid treatment are higher than when amino acid is given at rest [216]. Further studies are needed on how diet and exercise affect the way mitochondria and extracellular matrix interact.

## 5. Conclusions

Sarcopenia is one of the most severe geriatric syndromes. Under the trend of global aging, there is an urgent need to develop effective preventive and therapeutic measures. In the past decades, great progress has been made to elucidate the cellular and molecular mechanisms underlying the pathophysiology of this senile syndrome. The changes in skeletal muscle ECM are partially responsible for the worsened muscle function during the ageing process. And several aspects of age-related mitochondria alterations, including mtDNA damage, imbalance of dynamics, biogenesis decline, mitophagy impairment, and ROS overproduction, seem to contribute to the development and deterioration of musculoskeletal aging. Moreover, emerging evidence suggests a clear crosstalk between mitochondria, ROS, and ECM in different tissues and organs. However, the detailed impacts of those changes and crosstalk in aging pathobiology, especially in skeletal muscle, remain to be clarified. Further research is needed to provide deeper insight into the role of skeletal muscle ECM and mitochondria in sarcopenia, which may help with preventing and ameliorating prognoses.

## Data Availability

Data sharing is not applicable to this article as no new data were created or analyzed in this study.
